# Heat in Disease

**Published:** 1874-12

**Authors:** 


### HEAT IN DISEASE.

A writer in this city, whom we take to be Dr. Sayre or Dr. Hamilton, contributes several very interesting letters to the *Salt Lake Daily Herald*, in which health and hygienic topics of importance are very ably and entertainingly written. From a recent letter of this writer, we extract the following :

"A United States physician of great skill, was at one time at the head of the government hospital in Chili. For seven years he had been a victim to a most terrible form of disease known as *cystitis.* He had traversed Europe in search of relief. The most celebrated surgeons had assured him that a cure was impossible. Having exhausted the resources of the healing art he abandoned all hope of cure. In Chili there are certain hot baths supposed to possess marvelous virtues. Our suffering doctor paid them a visit from curiosity. There he met a wealthy Spaniard who told him that he annually visited the springs out of gratitude, as they had cured him, a few years before, of *cystitis* of fourteen years standing. Our doctor was beside himself with delight. He took the advice of the Spaniard and afterwards the baths. Fifteen days of soaking in water at 150° Fahrenheit, two hours at a time, and four hours of quiet repose and sweating afterwards, and then duty compelled his departure. He was not cured. He could not positively say that he was relieved. Yet ten days later, and no additional treatment, he was as well as any man ; and now, seven years afterwards, he assures me there has not been the smallest symptom of a return of the disease.

What cured him ? Well, he has a theory about it, and he has about concluded that it is a correct one. It took him some years and cost him some money to establish his theory; but he is ready to swear by it now. He had the water of the springs analyzed hot, cold and tepid, and it proved to be absolutely pure water with nothing whatever in solution. It was clear to the mind of this scientific

Allopath that he hadn't been cured by being boiled in a concoction of drugs.

But whenever he saw or heard of a poor creature suffering, as he had suffered, the torments of the damned with inflammation of the bladder, he advised him to try sweating, sweating to an extent which the average educated or uneducated medical personage would call dangerous. He told them to sweat just as long as they could endure it, and keep it up and make a business of it for not less than two weeks. What was the result ? In every case a cure.

And thus my friend was convinced that his disease was cured, not by medicine which his tissues had absorbed from the waters of the Chili springs, for they contained no such medicine, but rather by the prolonged and often repeated sweatings to which he has been subjected."

We happen to know something about this case, and can fully vouch for the truthfulness of the above statement. We have heretofore alluded to the value of heat as a remedial agent, and our opinion of its virtues is in no way weakened by the result of its use in the instance described above. In our October number we described the methods employed by Dr. E. P. Miller of 26th street, New York, whose establishment is probably the most widely known of any where heat is chiefly relied on,

